# Emergence of Within-Host SARS-CoV-2 Recombinant Genome After Coinfection by Gamma and Delta Variants: A Case Report

**DOI:** 10.3389/fpubh.2022.849978

**Published:** 2022-02-22

**Authors:** Ronaldo da Silva Francisco Junior, Luiz G. P. de Almeida, Alessandra P. Lamarca, Liliane Cavalcante, Yasmmin Martins, Alexandra L. Gerber, Ana Paula de C. Guimarães, Ricardo Barbosa Salviano, Fernanda Leitão dos Santos, Thiago Henrique de Oliveira, Isabelle Vasconcellos de Souza, Erika Martins de Carvalho, Mario Sergio Ribeiro, Silvia Carvalho, Flávio Dias da Silva, Marcio Henrique de Oliveira Garcia, Leandro Magalhães de Souza, Cristiane Gomes da Silva, Caio Luiz Pereira Ribeiro, Andréa Cony Cavalcanti, Claudia Maria Braga de Mello, Amilcar Tanuri, Ana Tereza R. Vasconcelos

**Affiliations:** ^1^Laboratório de Bioinformática, Laboratório Nacional de Computação Científica, Petrópolis, Brazil; ^2^Departamento de Genética, Instituto de Biologia, Universidade Federal do Rio de Janeiro, Rio de Janeiro, Brazil; ^3^Unidades de Apoio ao Diagnóstico da Covid-19, Rio de Janeiro, Brazil; ^4^Secretaria Estadual de Saúde do Rio de Janeiro, Rio de Janeiro, Brazil; ^5^Secretaria Municipal de Saúde Rio de Janeiro, Rio de Janeiro, Brazil; ^6^Laboratório Central de Saúde Pública Noel Nutels, Rio de Janeiro, Brazil

**Keywords:** recombination, SARS-CoV-2, COVID-19, iSNVs, coinfection

## Abstract

In this study, we report the first case of intra-host SARS-CoV-2 recombination during a coinfection by the variants of concern (VOC) AY.33 (Delta) and P.1 (Gamma) supported by sequencing reads harboring a mosaic of lineage-defining mutations. By using next-generation sequencing reads intersecting regions that simultaneously overlap lineage-defining mutations from Gamma and Delta, we were able to identify a total of six recombinant regions across the SARS-CoV-2 genome within a sample. Four of them mapped in the spike gene and two in the nucleocapsid gene. We detected mosaic reads harboring a combination of lineage-defining mutations from each VOC. To our knowledge, this is the first report of intra-host RNA-RNA recombination between two lineages of SARS-CoV-2, which can represent a threat to public health management during the COVID-19 pandemic due to the possibility of the emergence of viruses with recombinant phenotypes.

## Introduction

Since the first reports of patients coinfected by two genetically-distinct lineages of SARS-CoV-2 (1–7), the scientific community raised concerns about the recombination of intra-host viral RNA sequences as a possible mechanism underlying the emergence of novel variants. Indeed, this phenomenon occurs at a relatively high frequency among betacoronaviruses (8–10). In SARS-CoV-2, signals of ongoing recombination were observed in a few sequences deposited in GISAID and circulating in North America (11, 12). Both studies showed evidence of recombination between non-variants of concern. Given the emergence and widespread dispersion of variants of concern (VOC) harboring advantageous mutation able to alter the virus phenotype around the world, the possibility of recombination between them could represent a faster and more dangerous path to increase diversity facilitating the emergence of novel VOCs. The generation of recombinant genomes mainly requires (i) circulation of different lineages in the population, (ii) coinfection events, and (iii) intra-host recombination of the coinfected lineages. For this reason, we continuously monitored the presence of coinfected samples in the bam files of the samples originated from the state of Rio de Janeiro, Brazil. During the routine analysis across the 5,073 SARS-CoV-2 genomes generated by the Corona-ômica-RJ project (http://www.corona-omica.rj.lncc.br/#/), we identified the coinfection between Gamma and Delta reported next.

## Case Description

In late July, a 32-year-old male with mild flu-like symptoms of fever, headache, cough, fatigue, and sore throat was referred to the Santo Antônio de Pádua municipal health department in the northeastern region of the state of Rio de Janeiro, Brazil. After 2 days from the onset of symptoms, the diagnosis of COVID-19 was confirmed by the SARS-CoV-2 reverse transcriptase-polymerase chain reaction (RT-PCR) assay of a nasopharyngeal swab. Given the mild symptoms, no hospitalization was required and the patient was quarantined at home. No alterations in blood pressure or saturation were observed during the course of the disease. The patient did not report any comorbidities and had not been vaccinated by the time the sample was collected. The sample's relative quantification of the viral load (RQVL = 2^−ΔCT^) exhibited elevated values, with an RT-PCR cycle threshold (Ct) of 16.70 corresponding to the top 3% of samples with the highest viral load from Gamma variants in our database (*n* = 1,881). After 24 days from the symptoms onset, the patient presented the first negative RT-PCR test.

## Genomic Evidence of Coinfection and Recombination

Whole-genome sequencing analysis detected 73 intra-host single nucleotide variants (iSNVs) with allele frequency >5% and depth >100 reads. Of these, 26 were lineage-defining mutations exclusively found in the variant of concern (VOC) Gamma (P.1) and 32 were from Delta (AY.33; Supplementary Table 1). By using a hypergeometric distribution approach (6), we estimate an overall haplotype frequency of approximately 16 and 82% for Delta and Gamma, respectively. Nevertheless, two iSNVs characteristics of VOC Delta (ORF1ab: I1091V—AF = 94%; and ORF7b: T40I—AF = 70%) were found with a high frequency in our analysis, suggesting that some genomic copies from Gamma haplotype could also harbor the same mutations (Supplementary Table 1).

**Figure 1:**
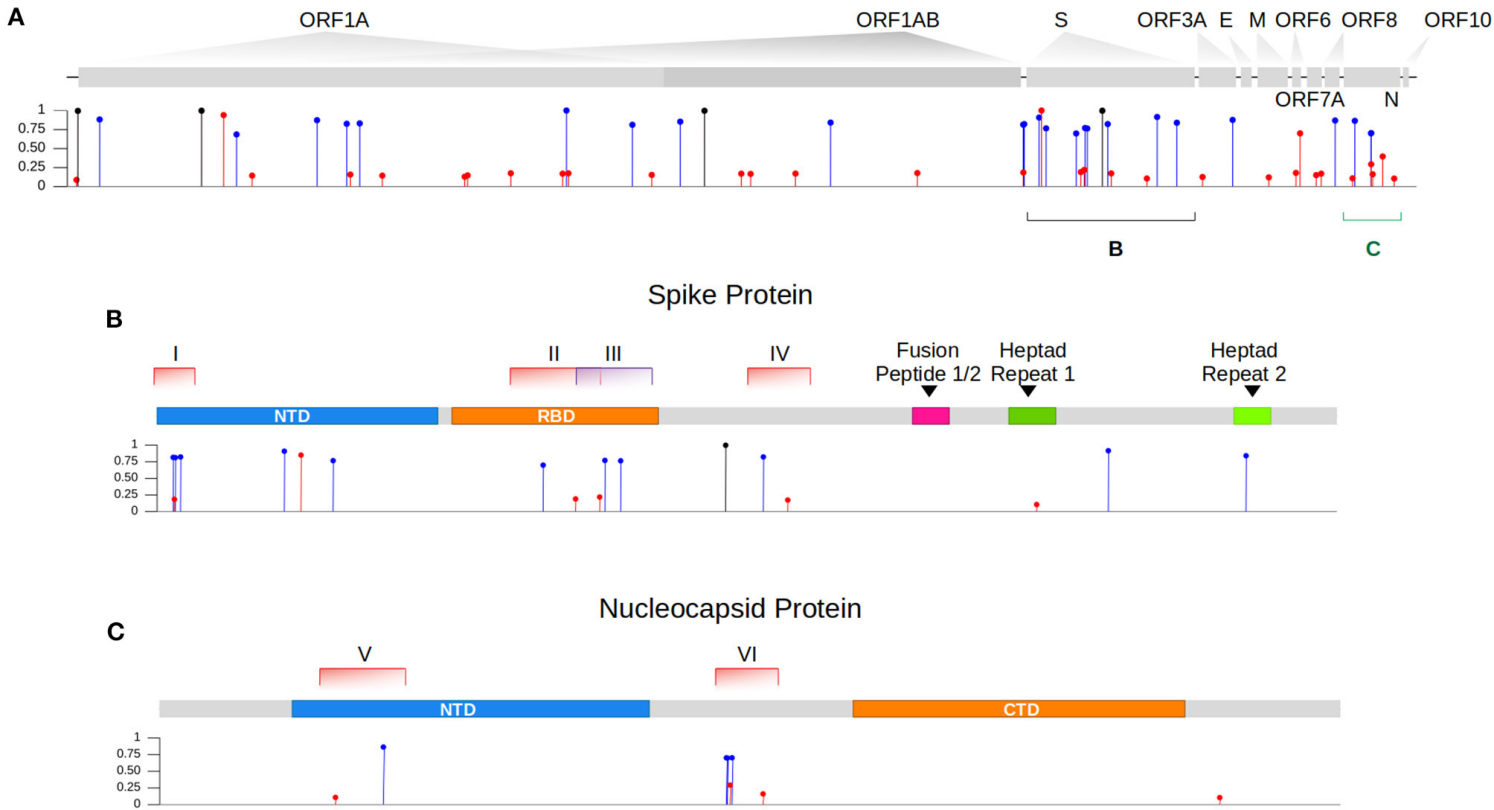
Intra-host single nucleotide variant frequencies evidence coinfection by two genetically distinct lineages. **(A)** Identification of iSNVs across the different genes of the SARS-CoV-2 genome within our sample. In blue and red, we showed the lineage-defining mutations from the variant Gamma and Delta, respectively. iSNVs represented in black are mutations commonly found in both lineages that descend from their ancestor B.1. **(B)** Zoom in gene S showing the location of each genomic window (I-IV) where recombination events were investigated. **(C)** Zoom in gene N showing the distribution of both selected regions (V and VI) across the gene.

We then sought to investigate the occurrence of within-host recombination events between the two lineages using next-generation sequencing. In theory, mutations characteristic of Gamma should be kept in different reads from those defining mutations of Delta. We divided the SARS-CoV-2 genome into windows with at least 100 nucleotides, which is smaller than the read length of the Illumina COVIDSeq Test (Illumina) sequencing kit used in this study. Next, we only selected windows that intersected at least two iSNVs, being one lineage-defining mutation from Gamma and the other from Delta. We aimed to detect unfragmented reads that covered the entire window harboring the iSNVs selected. A total of six recombinant candidate regions across the SARS-CoV-2 genome were found (see Supplementary Material), four mapped in the spike gene and two in the nucleocapsid gene (Table 1; Figure 1). We detected an average of two recombinant haplotypes supported by reads harboring a combination of the discriminating mutations from Gamma and Delta (Table 1; Supplementary Figures 1–6). In addition, the within-host recombinant sequences were formed by a single breakpoint event with a minimal and maximum interval of 15 and 75 nucleotides between two discriminate mutations where an event of recombination was observed, respectively.

**Table 1:** Evidence of emergence of recombinant haplotypes within a sample coinfected by Gamma and Delta variants with the mutations characteristic of Gamma and Delta are highlighted in blue and red, respectively.

**Region**	**Gene**	**Genomic interval**	**Number of bases**	**Number of defining mutations**	**Haplotypes**	**Number of reads**	**Protein Sequence**	**Classification**	**Amplicon ARTIC V3**	**Amplicon ARTIC V3 Coords**	**Coverage: mean (sd)**
I	S	21,614–21,638	24	4	TCAT	843	18F, 19T, 20N, 26S	Gamma	71	21386 - 21716	1185 (± 308)
					CGCC	167	18L, 19R, 20T, 26P	Delta			
					CGCT	42	18L, 19R, 20T, 26S	Recombinant			
					TCAC	28	18F, 19T, 20N, 26P	Recombinant			
II	S	22,917–23,012	95	3	TCA	234	452L, 478T, 484K	Gamma	76	22821 - 23189	1197 (±232)
					GAG	35	452R, 478K, 484E	Delta			
					TAG	36	452L, 478K, 484E	Recombinant			
					TCG	20	452L, 478T, 484E	Recombinant			
III	S	22,995–23,063	68	3	CAT	533	478T, 484K, 501Y	Gamma	76	22821 - 23189	1197 (± 232)
					AGA	104	478K, 484E, 501N	Delta			
					AGT	42	478K, 484E, 501Y	Recombinant			
					CGA	30	478T, 484E, 501N	Recombinant			
					CAA	25	478T, 484K, 501N	Recombinant			
IV	S	23,525–23,604	79	2	TC	694	655Y, 681P	Gamma	78	23466 - 23822	2066 (± 715)
					CG	148	655H, 681R	Delta			
					TG	44	655Y, 681R	Recombinant			
					CC	39	655H, 681P	Recombinant			
V	N	28,461–28,512	51	2	AG	950	63D, 80R	Gamma	94	28416 - 28756	1952 (±532)
					GC	73	63G, 80P	Delta			
					GG	50	63G, 80R	Recombinant			
					AC	33	63D, 80P	Recombinant			
VI	N	28,877–28,916	39	6	TCAACG	822	202C, 203K, 204R, 215G	Gamma	95	28699 - 29041	1290 (±223)
					AGTGGT	101	202T, 203M, 204G, 215C	Delta			
					AGTGGG	40	202T, 203M, 204G, 215G	Recombinant			
					TCAACT	43	202C, 203K, 204R, 215C	Recombinant			

From the six regions selected in our analysis, four overlapped the spike gene and two the nucleocapsid gene (Figure 1). All recombinant haplotypes were found at a low frequency compared to the reads supporting the sequences from each VOC (Table 1). Region I is located at the 5' region of the spike gene and includes three lineage-defining mutations from Gamma (S: L18F, T20N, and P26S) and one from Delta (S: T19R; Supplementary Figure 1). We identified two regions (II and III; Supplementary Figures 2, 3) overlapping the receptor-binding domain (RBD). Region II included two amino acid residue alterations from Delta (S: L452R, T478K) and one from Gamma (S: E484K; Supplementary Figure 2). The T478K and E484K mutations were also present in Region III (Supplementary Figure 3), which harbored a second alteration from Gamma (S: N501Y). This region was the only window where three different recombinant haplotypes were observed (Table 1; Supplementary Figure 3). The last region (IV) in the spike includes only one mutation from Gamma (S: H655Y) and one from Delta (S: 681R; Supplementary Figure 4). At the 5′ portion of the nucleocapsid gene, we selected a window (region V; Supplementary Figure 5) containing a single mutation from Delta (N: D63G) and from Gamma (N: P80R). Finally, the largest region selected (VI) was also mapped in N and included four mutations from Gamma (N: S202C, S202T, R203K, G204R) and two from Delta (R203M, G215C; Supplementary Figure 6).

The recombination in our sample was detected during the first reports of Delta in the city of Santo Antônio de Pádua and an ongoing replacement of the variant Gamma by the Delta in the state of Rio de Janeiro. Nevertheless, genomic surveillance data from the state of Rio de Janeiro indicated a frequency of 47% of sequences from Delta and 37% from Gamma (13) in July 2021. Thus, the epidemiological, geographic, and genomic data provide a conducive scenario to SARS-CoV-2 recombination.

## Discussion

The designation of VOC prioritizes genome sequences harboring genetic markers with demonstrated evidence for increasing virus transmissibility, virulence, or decreasing the effectiveness of control measures. In this context, the VOC Gamma and Delta were notable by causing an elevated number of Covid-19 cases and deaths in Brazil and worldwide, respectively. Both VOC are known to enhance SARS-CoV-2 transmissibility and induce an immune-escape response. Therefore, recombination between both sequences could represent the emergence of novel variants of concern with new phenotypic combinations. For example, we noticed the presence of the mutation N501Y, a lineage-defining mutation from Gamma, in a haplotype carrying predominantly mutations from Delta in Region III. This mutation is widely known to increase ACE2 binding (14). We also observed the emergence of recombinant haplotypes that lost advantageous alleles such as those from Gamma harboring 484E in region II, 501N in region III, and from Delta carrying 681P in region IV.

Homologous recombination in positive-sense RNA viruses is driven by a copy-choice replication mechanism with a switch from an RNA template by the RNA-polymerase complex during replication (15, 16). Thus, the recombinant haplotypes found across the different regions selected can represent multiple events of switches occurring within a cell or a few switches from RNA templates simultaneously occurring at multiple cells. These variations could be associated with a mechanism of escaping the host's immune response and should be further investigated for the development of antiviral therapy and vaccines. Although the number of reads intersecting the discriminating iSNVs in the recombinant haplotypes seems to be small, we also observed a huge number of reads covering each mutation separately (Supplementary Table 1; median read depth: 1947). Thus, the read coverage and minor allele frequency in each site suggest that these mutations were not caused by low mapping quality or miscalling variant issues.

The hypothesis that the recombination might be the product of laboratory contamination is highly discouraged due to the protocol used by us. All viral particles are inactivated before RNA extraction, which stops replication and, consequently, impairs recombination from happening due to sample contamination. In addition, the high efficiency of ARTIC pool of primers utilized in our library construction rule out the possibility of the recombinant fragment being made by the initial PCR amplification step. Moreover, the 100 nucleotide window used in our genome scan and the similarity of sequence breakpoints analyzed in different genome regions make it difficult to imagine that it can be produced by the PCR step used in our library construction. One alternative speculation about the presence of mosaic reads in our sample is that the sequences could be generated by reverse mutational processes that rescue the ancestral state of the Wuhan reference sequence in each haplotype. Nevertheless, to consider an elevated number of reverse mutations happening at the same time in a coinfected sample would exceed the rate of intra-host evolution previously observed (4, 17). Even if this high rate would be accepted as real, it is unlikely that these mutations would occur only in the “reverse direction” instead of generating a myriad of new substitutions.

Recent studies reported the detection of SARS-CoV-2 recombinant lineages circulating at a low frequency (11, 12, 18–21). Nevertheless, these studies were restricted to detecting the inter-host dissemination of genomes post-recombination events, which could be biased by spurious mutations generated due to library preparation, laboratory contamination, and unavailability of raw sequenced data. Our identification of a reliable recombination event between VOCs raises a red alert for the possible emergence of a new VOC in the future that combines advantageous mutations from independent lineages. Such an event could represent an evolutionary jump of SARS-CoV-2, immediately increasing the fitness of the VOC in a way that this hypothetical variant would fastly dominate current variants and generate a new wave worldwide (as currently seen with Omicron). While the recombination detected in this work involves mutations associated with fitness increase (L452R, E484K, N501Y), the absence of the recombinant sequences in SARS-CoV-2 samples was obtained posteriorly to this case suggests that it was not enough to supplant the circulating variants.

In summary, we report in this work the first case of intra-host SARS-CoV-2 recombination during a coinfection by the VOC Delta (AY.33) and Gamma (P.1) supported by sequencing reads harboring a mosaic of lineage-defining mutations as well as putative sequences breakpoints. We did not detect the circulation of the recombinant sequences in the state, so far. Nevertheless, such identification deeply depends on the access to raw read data from the state of Rio de Janeiro and other locations across the world. The faster detection and surveillance of recombinant SARS-CoV-2 genomes require an increase in the number of sequencing, specially in countries with an elevated number of cases, and rapid deposition of both assembled genomes and raw reads in public databases.

## Data Availability Statement

The datasets presented in this study can be found in online repositories. The names of the repository/repositories and accession number(s) can be found below: https://www.ncbi.nlm.nih.gov/, PRJNA774631.

## Ethics Statement

The studies involving human participants were reviewed and approved by Ethics Committee (30161620.0.1001.5257 and 34025020.0.0000.5257). Written informed consent for participation was not required for this study in accordance with the national legislation and the institutional requirements.

## Author Contributions

ATRV: conceptualization. LC, ALG, APCG, RBS, FLS, and THO: DNA extraction and sequencing. IVS, EMC, MSR, SC, FDS, MHOG, LMS, CGS, CLPR, ACC, and CMBM: project administration. RSFJ, LGPA, APL, and YM: formal analysis. RSFJ and LGPA: data curation. ATRV: funding. RSFJ, APL, LGPA, and ATRV: writing – original draft. AT and ATRV: writing – review and editing. All authors contributed to the article and approved the submitted version.

## Funding

This work was developed in the frameworks of Corona-ômica-RSFJ (FAPERJ = E-26/210.179/2020 and E-26/211.107/2021). ATRV is supported by CNPq (303170/2017-4) and FAPERJ (E-26/202.903/20). AT is supported by FAPERJ E-26/010.002434/2019 and E-26/210.178/2020. RSFJ is a recipient of a graduate fellowship from CNPq. APL is granted a post-doctoral scholarship (DTI-A) from CNPq. We acknowledge the support from the Rede Corona-ômica BR MCTI/FINEP affiliated to RedeVírus/MCTI (FINEP 01.20.0029.000462/20, CNPq 404096/2020-4).

## Conflict of Interest

The authors declare that the research was conducted in the absence of any commercial or financial relationships that could be construed as a potential conflict of interest.

## Publisher's Note

All claims expressed in this article are solely those of the authors and do not necessarily represent those of their affiliated organizations, or those of the publisher, the editors and the reviewers. Any product that may be evaluated in this article, or claim that may be made by its manufacturer, is not guaranteed or endorsed by the publisher.
