# Supplementary material for: Emergence of Within-Host SARS-CoV-2 Recombinant Genome After Coinfection by Gamma and Delta Variants: A Case Report
Source: Front Public Health. 2022 Feb 22;10:849978. doi: 10.3389/fpubh.2022.849978 (PMC8902039; doi:10.3389/fpubh.2022.849978)
Supplement: Supplementary file 2 [file Data_Sheet_1.DOCX]

**Supplementary Material**

Emergence of within-host SARS-CoV-2 recombinant genome after coinfection by Gamma and Delta variants: a case report

Ronaldo da Silva Francisco Junior¹, Luiz G P de Almeida¹, Alessandra P Lamarca¹, Liliane Cavalcante¹, Yasmmin Martins¹, Alexandra L Gerber¹, Ana Paula de C Guimarães¹, Ricardo Barbosa Salviano², Fernanda Leitão dos Santos², Thiago Henrique de Oliveira², Isabelle Vasconcellos de Souza^3^, Erika Martins de Carvalho^3^, Mario Sergio Ribeiro^4^, Silvia Carvalho^4^, Flávio Dias da Silva^5^, Marcio Henrique de Oliveira Garcia^5^, Leandro Magalhães de Souza^6^, Cristiane Gomes Da Silva^6^, Caio Luiz Pereira Ribeiro^5^, Andréa Cony Cavalcanti^6^, Claudia Maria Braga de Mello^4^, Amilcar Tanuri², Ana Tereza R Vasconcelos¹*

*Corresponding author: atrv@lncc.br

^1^ Laboratório de Bioinformática, Laboratório Nacional de Computação Científica, Petrópolis, Brazil.

^2^ Departamento de Genética, Instituto de Biologia, Universidade Federal do Rio de Janeiro, Rio de Janeiro, Brazil.

^3^ Unidades de Apoio ao Diagnóstico da Covid-19, Rio de Janeiro, Brazil.

^4^ Secretaria Estadual de Saúde do Rio de Janeiro, Rio de Janeiro, Brazil.

^5^ Secretaria Municipal de Saúde Rio de Janeiro, Rio de Janeiro, Brazil

^6^ Laboratório Central de Saúde Pública Noel Nutels, Rio de Janeiro, Brazil.

**Supplementary Methods**

**Sample collection, RNA extraction, and sequencing**

The nasopharyngeal swab for SARS-CoV-2 RT-PCR test was collected and referred to the Noel Nutels Central Laboratory (LACEN-RJ). The viral RNA was extracted at the Molecular Virology Laboratory (LVM-UFRJ) with MagMAX Viral / Pathogen Nucleic Acid Isolation kit and KingFisher automatic platform. Annealing of cDNA was conducted with 8,5 µl of the viral RNA extracted. Total RNA from SARS-CoV-2 positive samples was converted to cDNA using the SuperScript IV First-Strand Synthesis System (Thermo Fisher Scientific, USA). We used Artic Network protocol (https://artic.network/ncov-2019) with the SARS-CoV-2 primer scheme (V3) for viral whole-genome amplification. Next-generation sequencing steps including library preparation were performed at the DFA/LNCC Genomics Unit with Illumina COVIDSeq Test (Illumina), according to the manufacturer’s protocol. Purification was then conducted using 5 µl of each library combined, and the TapeStation (Agilent) system was used for quality control. NextSeq 500/550 Mid Output Kit v2.5 (300 Cycles) was used to generate reads of 2x149 bp in the NextSeq (Illumina).

**Bioinformatic analysis**

Initially, adapter sequences generated next-generation sequencing were removed using cutadapt v.3.0 [(1)](https://paperpile.com/c/zYZuTx/nn252). We then performed the short read mapping to the SARS-CoV-2 reference genome (NC_045512.2) using bowtie2 v.2.2.1 [(2)](https://paperpile.com/c/zYZuTx/o99t) and bwa v.0.7.12-r1039 [(3)](https://paperpile.com/c/zYZuTx/91FFn). The post-processing steps and variant calling were performed independently for each output file. SAM format files were converted to BAM and sorted using samtools v.1.11 [(4)](https://paperpile.com/c/zYZuTx/AJ1dO). Next, duplicate reads were marked and removed using MarkDuplicates from Picard (https://broadinstitute.github.io/picard/). We performed the variant calling using HaplotypeCaller from GATK v.4.1.7.0 [(5)](https://paperpile.com/c/zYZuTx/HZnrF) and LoFreq v2.1.5 [(6)](https://paperpile.com/c/zYZuTx/9lsZQ). Next, iSNVs identified were annotated with snpEff/SnpSift v5.0e [(7)](https://paperpile.com/c/zYZuTx/gQ8w0).

We determined the candidate within-host lineages according to the lineage-defining mutations of each lineage, mutation frequencies, and concentration. This strategy uses as default a hypergeometric distribution approach to filtered coinfection events [(8)](https://paperpile.com/c/zYZuTx/SJnN). To confirm such inference, RegressHaplo was applied as an alternative method [(9)](https://paperpile.com/c/zYZuTx/zLqJZ). We retrieved the mosaic reads covering the genomic windows selected using sam2tsv [(10)](https://paperpile.com/c/zYZuTx/7jm6e). IGV v.2.9.4 was used to visualize the mapping in the target regions. Finally, consensus sequences were classified according to the Pango Lineage classification using the PangoLEARN model database v3.5.3.

**Supplementary Figures**


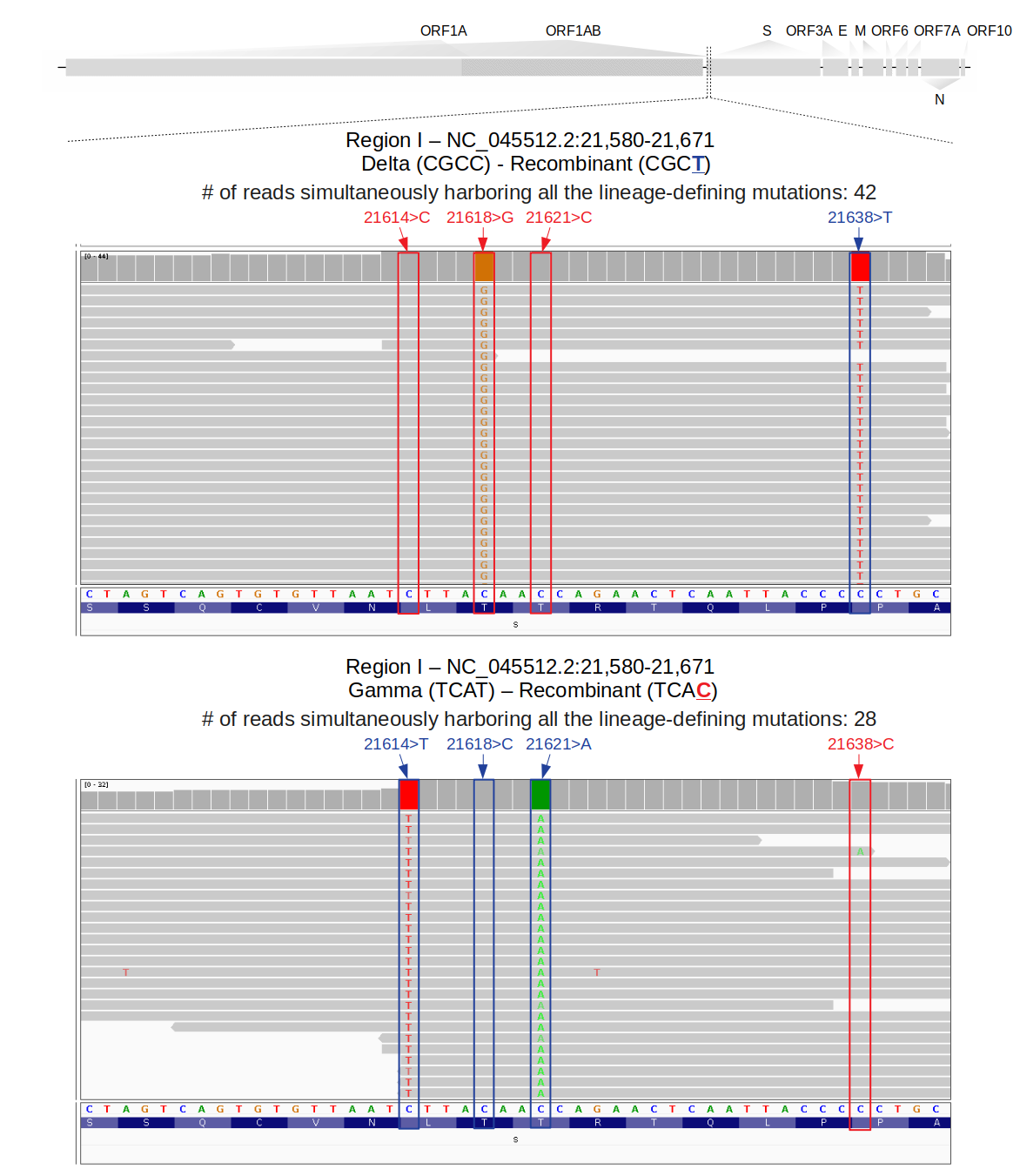


**Figure S1. Visualization of sequenced reads covering recombinant haplotypes found in he region I.** Screenshot of some mosaic reads found in region I as proof of within-host recombination. Blue and red rectangles represent lineage-defining sites where an alternative allele characteristic of Gamma and Delta variants was found, respectively.


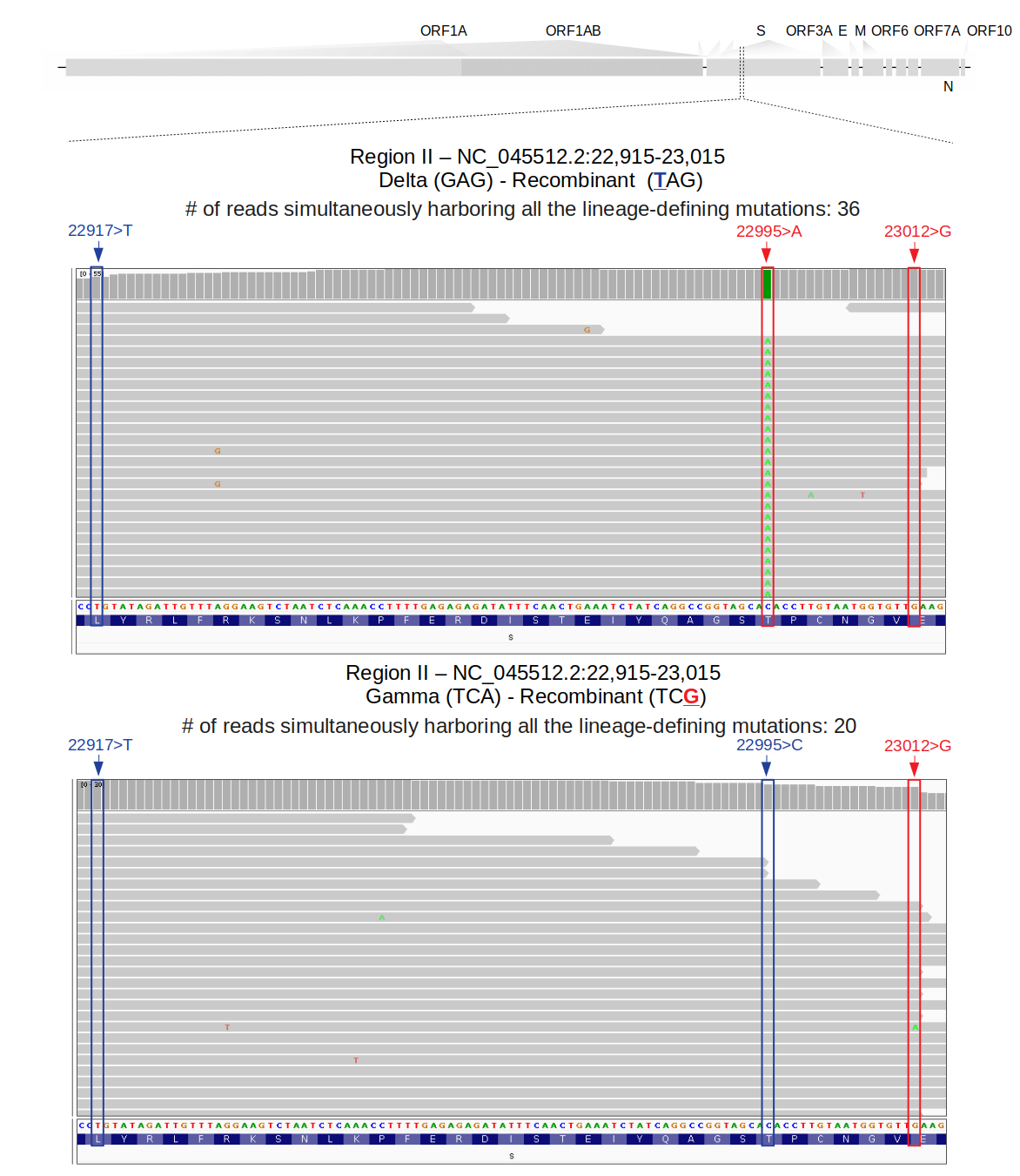


**Figure S2. Visualization of sequenced reads covering recombinant haplotypes found in region II.** Screenshot of some mosaic reads found in region II as proof of within-host recombination. Blue and red rectangles represent lineage-defining sites where an alternative allele characteristic of Gamma and Delta variants was found, respectively.


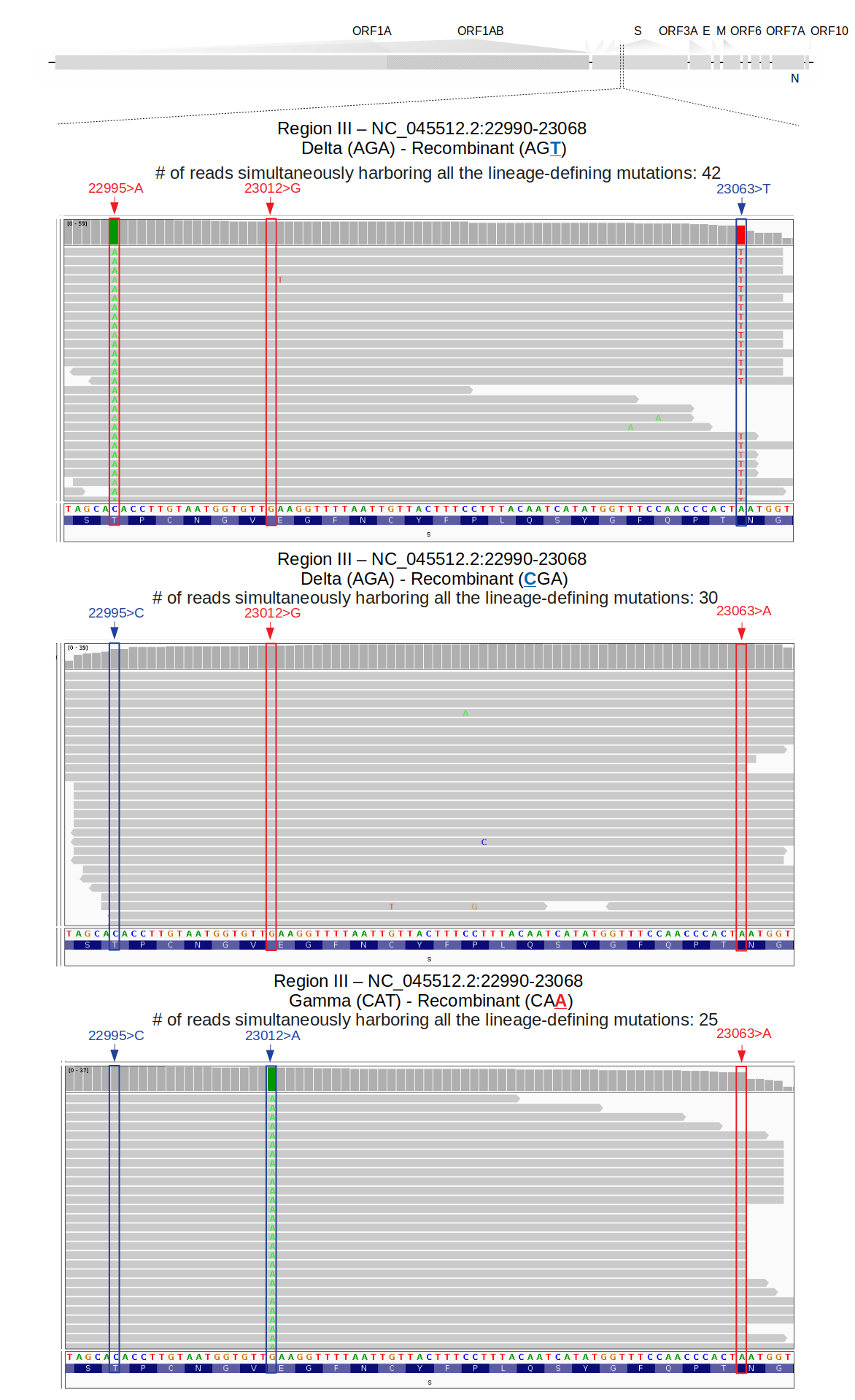


**Figure S3. Visualization of sequenced reads covering recombinant haplotypes found in region III.** Screenshot of some mosaic reads found in region III as proof of within-host recombination. Blue and red rectangles represent lineage-defining sites where an alternative allele characteristic of Gamma and Delta variants was found, respectively.


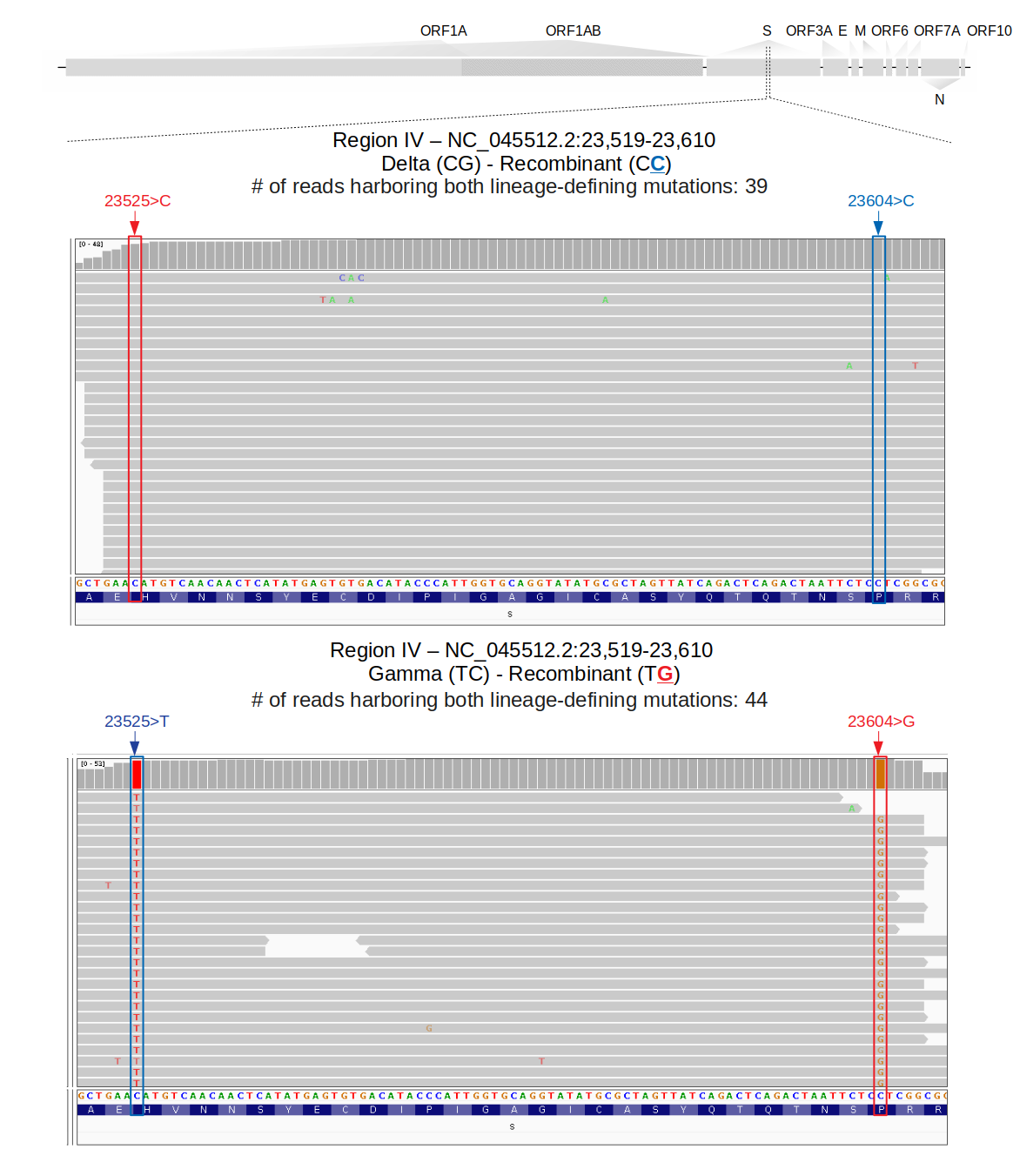


**Figure S4. Visualization of sequenced reads covering recombinant haplotypes found in region IV.** Screenshot of some mosaic reads found in region IV as proof of within-host recombination. Blue and red rectangles represent lineage-defining sites where an alternative allele characteristic of Gamma and Delta variants was found, respectively.


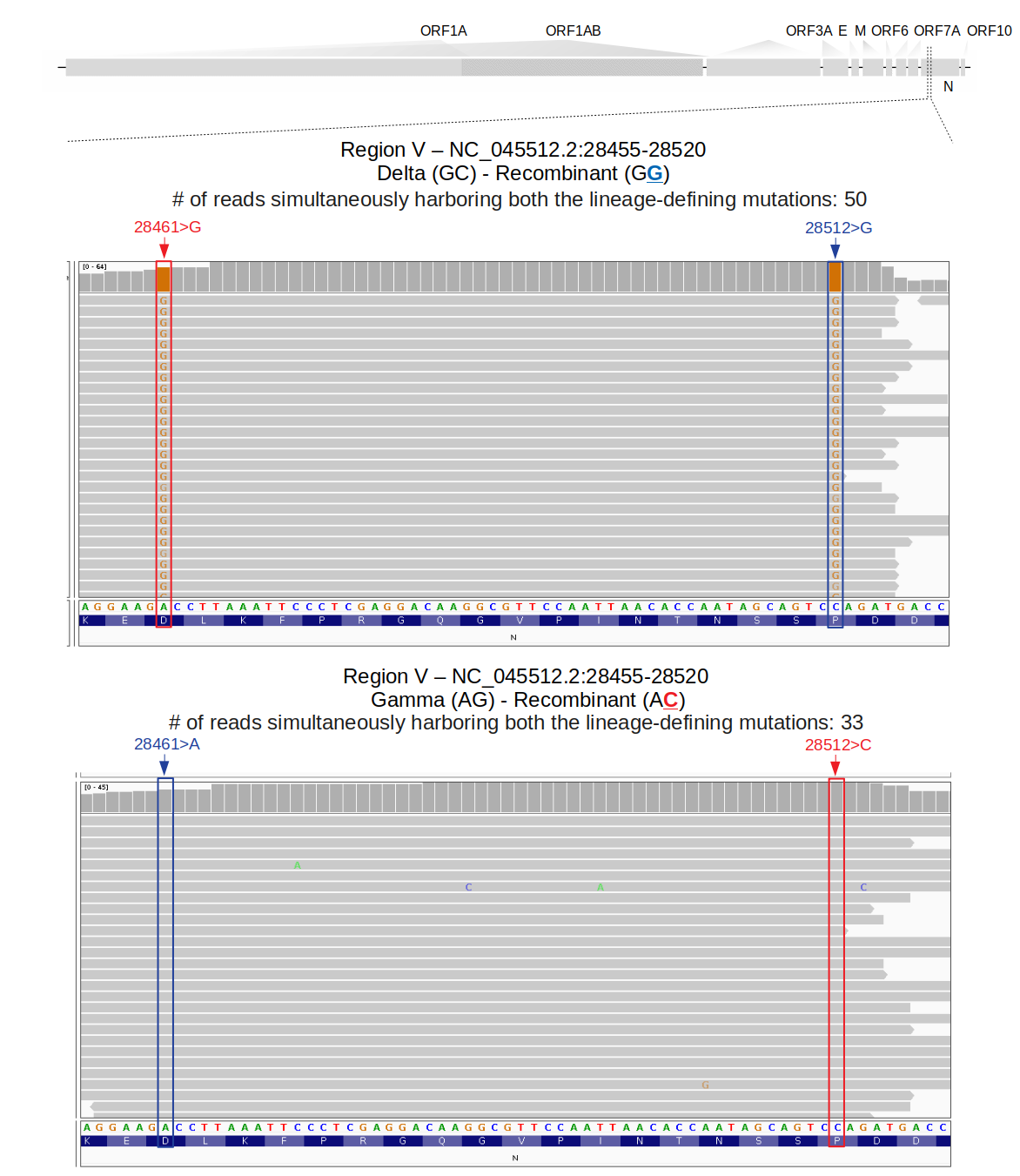


**Figure S5. Visualization of sequenced reads covering recombinant haplotypes found in region V.** Screenshot of some mosaic reads found in region V as proof of within-host recombination. Blue and red rectangles represent lineage-defining sites where an alternative allele characteristic of Gamma and Delta variants was found, respectively.


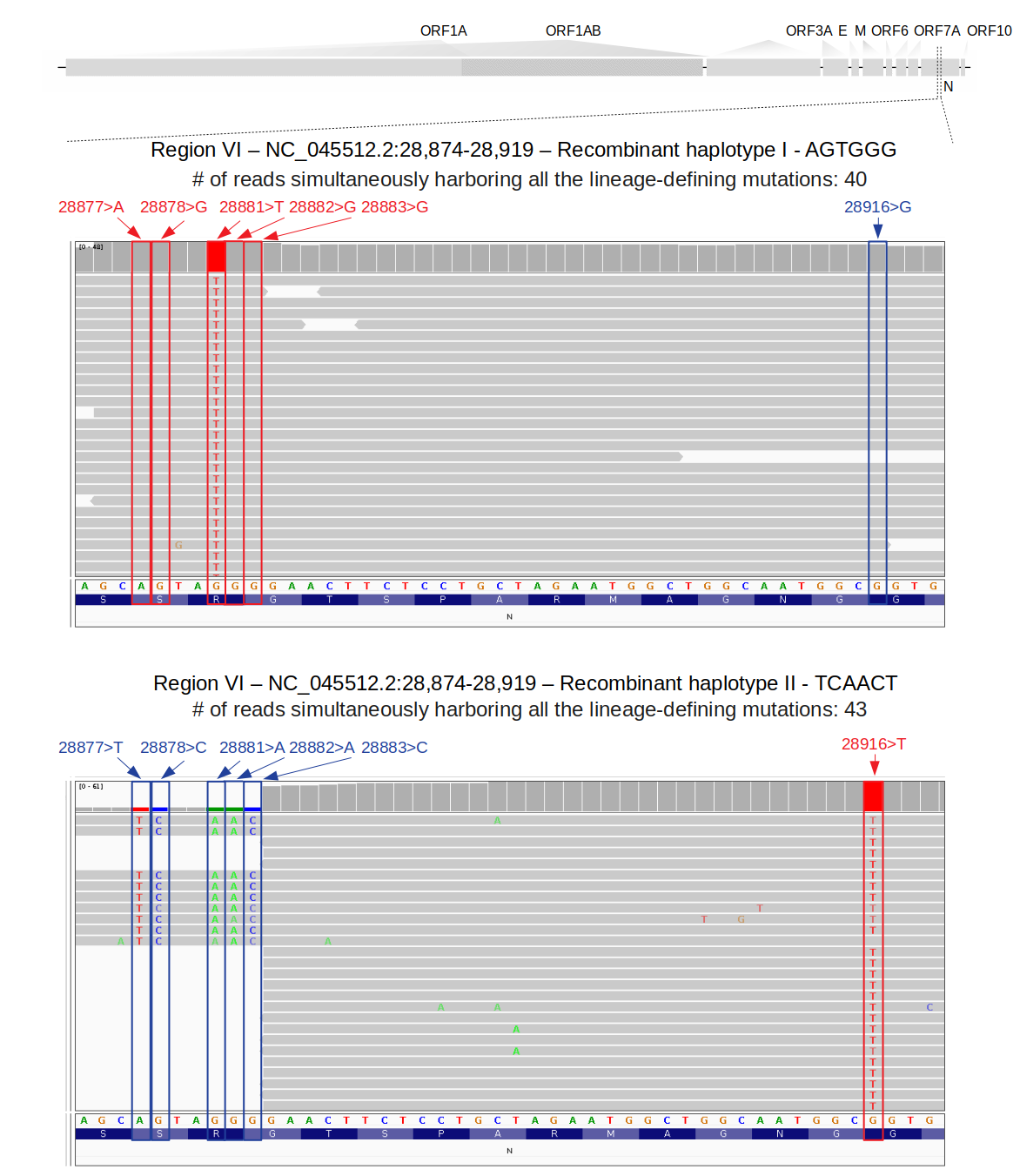


**Figure S6. Visualization of sequenced reads covering recombinant haplotypes found in region VI.** Screenshot of some mosaic reads found in region VI as proof of within-host recombination. Blue and red rectangles represent lineage-defining sites where an alternative allele characteristic of Gamma and Delta variants was found, respectively. Despite the limited number of reads shown, more fragments were found in each region.

**References**

1. [Martin M. Cutadapt removes adapter sequences from high-throughput sequencing reads. *EMBnet J* (2011) 17:10.](http://paperpile.com/b/zYZuTx/nn252)

2. [Langmead B, Salzberg SL. Fast gapped-read alignment with Bowtie 2. *Nat Methods* (2012) 9:357–359.](http://paperpile.com/b/zYZuTx/o99t)

3. [Li H, Durbin R. Fast and accurate short read alignment with Burrows-Wheeler transform. *Bioinformatics* (2009) 25:1754–1760.](http://paperpile.com/b/zYZuTx/91FFn)

4. [Li H, Handsaker B, Wysoker A, Fennell T, Ruan J, Homer N, Marth G, Abecasis G, Durbin R, 1000 Genome Project Data Processing Subgroup. The Sequence Alignment/Map format and SAMtools. *Bioinformatics* (2009) 25:2078–2079. doi:](http://paperpile.com/b/zYZuTx/AJ1dO)[10.1093/bioinformatics/btp352](http://dx.doi.org/10.1093/bioinformatics/btp352)

5. [DePristo MA, Banks E, Poplin R, Garimella KV, Maguire JR, Hartl C, Philippakis AA, del Angel G, Rivas MA, Hanna M, et al. A framework for variation discovery and genotyping using next-generation DNA sequencing data. *Nature Genetics* (2011) 43:491–498. doi:](http://paperpile.com/b/zYZuTx/HZnrF)[10.1038/ng.806](http://dx.doi.org/10.1038/ng.806)

6. [Wilm A, Aw PPK, Bertrand D, Yeo GHT, Ong SH, Wong CH, Khor CC, Petric R, Hibberd ML, Nagarajan N. LoFreq: a sequence-quality aware, ultra-sensitive variant caller for uncovering cell-population heterogeneity from high-throughput sequencing datasets. *Nucleic Acids Research* (2012) 40:11189–11201. doi:](http://paperpile.com/b/zYZuTx/9lsZQ)[10.1093/nar/gks918](http://dx.doi.org/10.1093/nar/gks918)

7. [Cingolani P, Platts A, Wang LL, Coon M, Nguyen T, Wang L, Land SJ, Lu X, Ruden DM. A program for annotating and predicting the effects of single nucleotide polymorphisms, SnpEff. *Fly* (2012) 6:80–92. doi:](http://paperpile.com/b/zYZuTx/gQ8w0)[10.4161/fly.19695](http://dx.doi.org/10.4161/fly.19695)

8. [Zhou H-Y, Cheng Y-X, Xu L, Li J-Y, Tao C-Y, Ji C-Y, Han N, Yang R, Li Y, Wu A. Genomic evidence for divergent co-infections of SARS-CoV-2 lineages. *BioRxiv* (2021) doi:](http://paperpile.com/b/zYZuTx/SJnN)[10.1101/2021.09.03.458951](http://dx.doi.org/10.1101/2021.09.03.458951)

9. [Leviyang S, Griva I, Ita S, Johnson WE. A penalized regression approach to haplotype reconstruction of viral populations arising in early HIV/SIV infection. *Bioinformatics* (2017) 33:2455–2463.](http://paperpile.com/b/zYZuTx/zLqJZ)

10. [Lindenbaum P. JVarkit: java-based utilities for Bioinformatics. (2015) doi:](http://paperpile.com/b/zYZuTx/7jm6e)[10.6084/m9.figshare.1425030.v1](http://dx.doi.org/10.6084/m9.figshare.1425030.v1)
